# Mitochondrial and ribosomal markers in the identification of nematodes of clinical and veterinary importance

**DOI:** 10.1186/s13071-023-06113-4

**Published:** 2024-02-20

**Authors:** María José Mejías-Alpízar, Catalina Porras-Silesky, Esteban José Rodríguez, Joban Quesada, María Paula Alfaro-Segura, Joby Robleto-Quesada, Ricardo Gutiérrez, Alicia Rojas

**Affiliations:** 1 Laboratorio de Helmintología, Departamento de Parasitología, Facultad de Microbiología, University of Costa Rica, San José, Costa Rica; 2 Centro de Investigación en Enfermedades Tropicales, University of Costa Rica, San José, Costa Rica; 3 National Reference Center for Bacteriology, Costa Rican Institute for Research and Teaching in Nutrition and Health (INCIENSA), Tres Rios, Costa Rica; 4 Ross University School of Veterinary Medicine, West Farm, Basseterre, Saint Kitts and Nevis

**Keywords:** Molecular diagnosis, Phylogenetic analysis, Mitochondrial markers, Ribosomal markers, Ascarididae, Ancylostomatidae, Onchocercidae

## Abstract

**Background:**

Nematodes of the Ascarididae, Ancylostomatidae and Onchocercidae families are parasites of human and veterinary importance causing infections with high prevalence worldwide. Molecular tools have significantly improved the diagnosis of these helminthiases, but the selection of genetic markers for PCR or metabarcoding purposes is often challenging because of the resolution these may show.

**Methods:**

Nuclear 18S rRNA, internal transcribed spacers 1 (ITS-1) and 2 (ITS-2), mitochondrial gene cytochrome oxidase 1 (*cox*1) and mitochondrial rRNA genes 12S and 16S loci were studied for 30 species of the mentioned families. Accordingly, their phylogenetic interspecies resolution, pairwise nucleotide p-distances and sequence availability in GenBank were analyzed.

**Results:**

The 18S rRNA showed the least interspecies resolution since separate species of the *Ascaris*, *Mansonella*, *Toxocara* or *Ancylostoma* genus were intermixed in phylogenetic trees as opposed to the ITS-1, ITS-2, *cox*1, 12S and 16S loci. Moreover, pairwise nucleotide p-distances were significantly different in the 18S compared to the other loci, with an average of 99.1 ± 0.1%, 99.8 ± 0.1% and 98.8 ± 0.9% for the Ascarididae, Ancylostomatidae and Onchocercidae families, respectively. However, ITS-1 and ITS-2 average pairwise nucleotide p-distances in the three families ranged from 72.7% to 87.3%, and the *cox*1, 12S and 16S ranged from 86.4% to 90.4%. Additionally, 2491 *cox*1 sequences were retrieved from the 30 analyzed species in GenBank, whereas 212, 1082, 994, 428 and 143 sequences could be obtained from the 18S, ITS-1, ITS-2, 12S and 16S markers, respectively.

**Conclusions:**

The use of the *cox*1 gene is recommended because of the high interspecies resolution and the large number of sequences available in databases. Importantly, confirmation of the identity of an unknown specimen should always be complemented with the careful morphological examination of worms and the analysis of other markers used for specific parasitic groups.

**Graphical Abstract:**

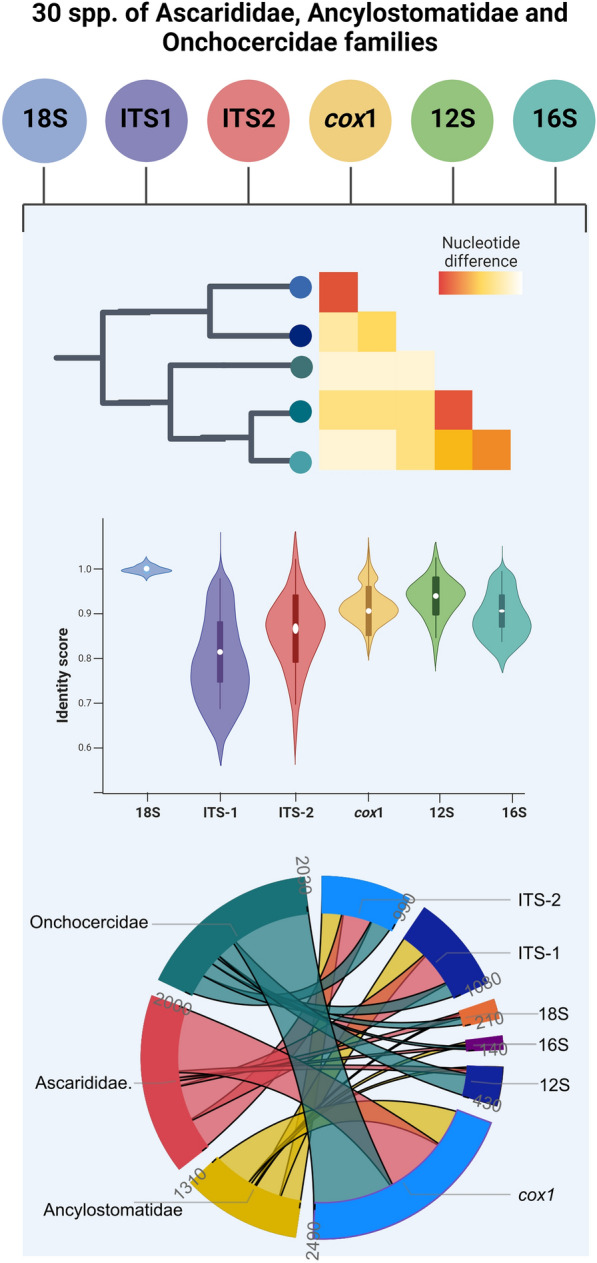

**Supplementary Information:**

The online version contains supplementary material available at 10.1186/s13071-023-06113-4.

## Background

Nematodes of the families Ascarididae, Ancylostomatidae and Onchocercidae are parasites of human and veterinary importance causing infections with high prevalence worldwide [1–3]. Many of the infections produced in humans are cataloged as tropical neglected diseases, associated to areas with low socioeconomic index and poor access to diagnostic tools and treatment [4, 5].

Members of the families Ascarididae, Ancylostomatidae and Onchocercidae may cause clinical manifestations in virtually all known mammal species, whom get infected by the intake of contaminated water or food, contact with soil or an intermediate host [6]. For instance, the human ascarid *Ascaris lumbricoides* is the most common human nematode reported worldwide, with up to 1.2 billion estimated people infected [7]. In the veterinary field, *Parascaris equorum* and *Baylisascaris procyonis* produce intestinal infections in equines and raccoons [8], respectively, whereas *Toxocara canis* generates infections in > 100 million dogs worldwide [9] and can produce a zoonotic disease in humans, known as visceral, ocular or cerebral larva migrans with an estimated global human seroprevalence of 19% [9, 10]. Furthermore, blood-sucking hookworms like *Necator americanus* and *Ancylostoma* spp. (Ancylostomatidae family) are responsible for 740 million people infected worldwide causing intestinal bleeding and chronic ferropenic anemia [4]. Finally, filarioids such as *Wuchereria bancrofti*, *Brugia malayi*, *Onchocerca* spp. and *Dirofilaria* spp. are vector-borne heteroxenous parasites of the family Onchocercidae with worldwide distribution that affect thousands of humans, companion animals, livestock and wildlife [5].

Sensitive diagnostic tools are essential to improve clinical detection and treatment in animal and human hosts. However, traditional parasitological methods of diagnosis usually show low sensitivity for detecting and identifying species [3]. For instance, although direct microscopic observation of stool samples and concentration methods like Kato-Katz, Sheather or Ritchie detect ascarid and hookworm eggs and larvae using unsophisticated equipment and inexpensive reagents [11], these methods are negatively affected by low parasitic loads, intermittent excretion of eggs and uneven distribution of eggs in stool samples, significantly affecting their sensitivity [12]. Similarly, the diagnosis of filariasis is based predominantly on the microscopic detection of circulating microfilariae in blood, either by direct blood smears or Knott's modified test, or the recognition of the worm antigens using immunochromatographic tests. However, false-negative results are common in low parasitemia, single-sex or mixed infections. To alleviate these limitations of classical methods in parasitology, molecular methods with higher sensitivity and specificity have been designed [13, 14].

Molecular tools have improved the diagnosis of infections and facilitated taxa differentiation and phylogenetic analysis of worms of the Ascarididae, Ancylostomatidae and Onchocercidae families [14, 15]. Mitochondrial genes such as cytochrome c oxidase subunit 1 (*cox*1), cytochrome b (*cyt*b), and the NADH dehydrogenase subunit 1 (*nad*1), and mitochondrial rRNA 12S and 16S are widely used to determine lower taxonomic levels (i.e. identification to genus and species-level) and resolve species-level phylogenies because of their high degree of sequence variation [16]. Similarly, internal transcribed spacers (ITS) 1 and 2 of the ribosomal nuclear DNA also show a high degree of sequence variation, promoting their use as markers for species differentiation [16]. Contrarily, the 18S nuclear rRNA gene is highly conserved within species and has low inter-species variability but has been employed for characterizing nematode communities at higher taxonomic levels [16]. Altogether, the variability of each genetic marker offers different resolution for phylogenetic analyses and practicability for diagnosis of nematodes [15].

Some challenges remain unsolved in the genetic analysis of nematodes. For instance, species hybrids have been initially interpreted as sequencing artifacts when discordances are detected during phylogenetic analyses using nuclear and mitochondrial genes [17]. Moreover, cryptic diversity identification in morphologically undistinguishable specimens relies on nucleotide differences among taxa and sequence availability in databases [18]. Therefore, cryptic diversity is accurately detected only when enough sequences are compared and when appropriate nuclear, ribosomal or mitochondrial markers are employed.

The aim of this study was to analyze the resolution and performance of six different ribosomal and mitochondrial loci (18S, ITS-1, ITS-2, *cox*1, 12S and 16S) for the interspecies differentiation of nematodes of the families Ascarididae, Ancylostomatidae and Onchocercidae of human and veterinary importance. Importantly, we estimated the number of available sequences on databases for each species included in the study to contextualize their usefulness in the clinical and research scenario. In this way, we evaluated the practicality of ribosomal and mitochondrial markers for the identification of worms as well as their ease of analysis and taxonomic resolution.

## Methods

**Fig. 1:**
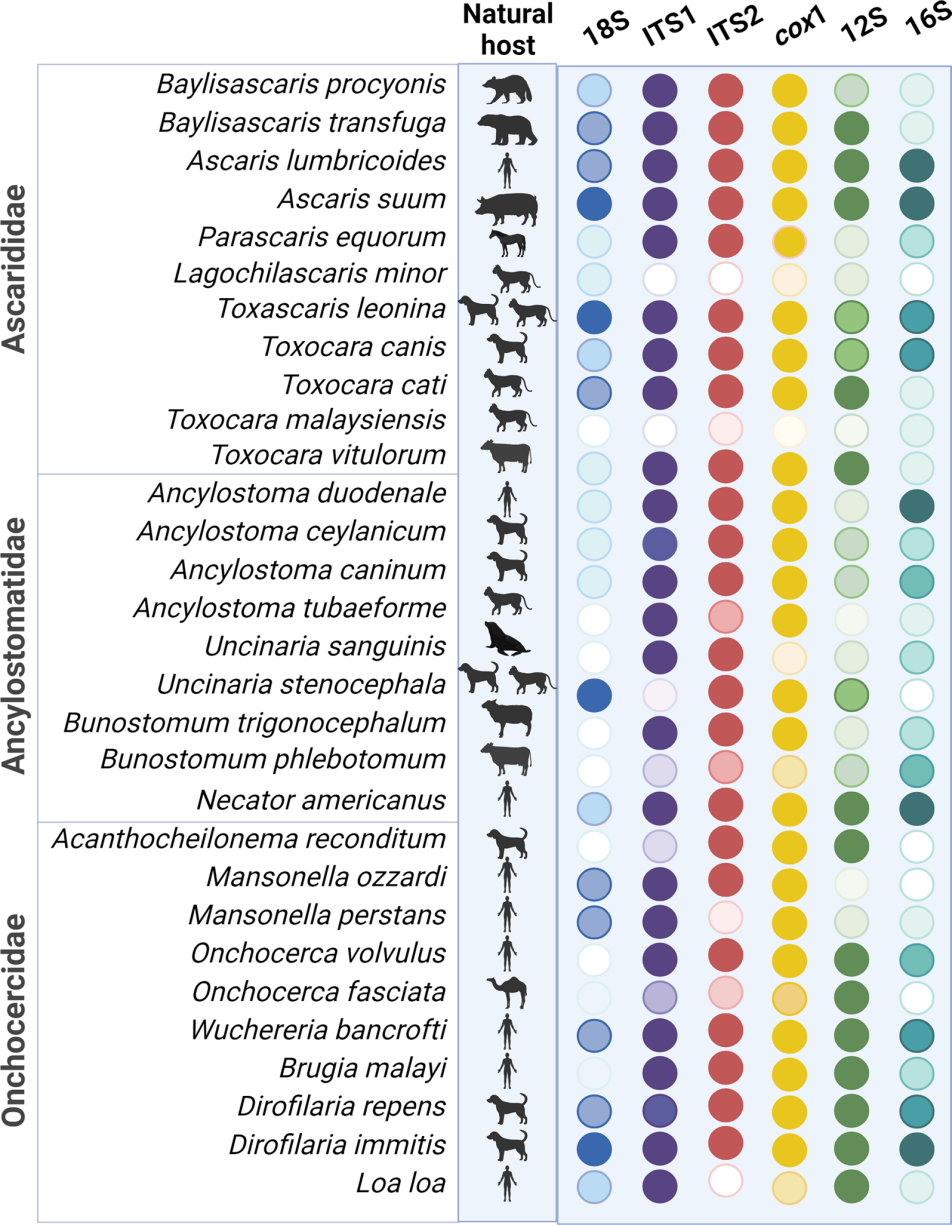
Ribosomal and mitochondrial sequences used for the phylogenetic analysis of 30 species of the Ascarididae, Ancylostomatidae and Onchocercidae families. The number of sequences is color coded from darkest (*n* = 5) to lightest (*n* = 0) according to the marker: 18S in blue, ITS1 in purple, ITS2 in red, *cox*1 in yellow, 12S in green and 16S in turquoise. This figure was created using Biorender.com

Six genetic markers from representative nematode species of medical and veterinary importance of the families Ascarididae, Onchocercidae and Ancylostomatidae were analyzed in this study (Fig. 1). Accordingly, five representative full-length or partial sequences of > 300 bp nuclear ribosomal regions ITS-1, ITS-2 and 18S rRNA and mitochondrial genes *cox*1, 12S and 16S rRNA were downloaded from the NCBI database (https://www.ncbi.nlm.nih.gov/) and aligned in MEGA X using the MUSCLE tool. Chosen sequences had to overlap with the consensus sequence block in at least 80% of length of the block to be included in the analysis. To take into account intraspecies variability, up to five sequences belonging to different hosts or geographical locations were included in the analysis. The accession numbers of the sequences included in this study are available in Additional file 1: Table S1.

The best nucleotide substitution model for each loci and family was computed with jModelTest software [19] based on the Bayesian inference criterion calculations (Additional file 2: Table S2). Bayesian inference (BI) phylogenetic trees were constructed using the BEAST2 package (https://www.beast2.org/) with 10^8^ MCMCs and a tree burn-in percentage of 10%. Then, the program Interactive Tree of Life (iTOL) (https://itol.embl.de/) was used to edit the resulting trees. Afterwards, we elaborated a color-coded matrix based on sequence pairwise identity using the program Sequence Demarcation Tool version 1.2 (SDTv1.2) [20], with alignments as input files and removing the option for calculating a neighbor-joining tree.

Violin plots of genetic p-distances were constructed using the R package ggplot2 [21]. Pairwise nucleotide p- distances were calculated in MEGA X. Data and script used for this purpose are available in the Additional file 3. The Gaussian distribution of data was verified using the Kolmogorov-Smirnov test to determine significance between pairwise p-distance values between markers. Then, a Kruskal-Wallis analysis for non-parametric data was performed followed by Dunn’s multiple comparison test, and significance was considered when *P* < 0.01. These analyses were run in GraphPad Prism version 10.

### Sequence availability

The total number of sequences from the abovementioned species and genetic markers were calculated using the NCBI database and BLAST tool (https://blast.ncbi.nlm.nih.gov/Blast.cgi). The search was done to assess the practicality of each marker in the context of a diagnostic challenge. BLAST search was done with a complete sequence for each species. If there was no reference sequence for a particular locus, the longest sequence was selected. To retrieve all sequences available in the NCBI database, all sequences obtained from the blastn search of the reference sequences were downloaded. In addition, a manual search using key words for each genetic marker and species was also performed to retrieve more divergent sequences. Afterwards, we proofread all sequences and maintained only those with a length > 300 bp and > 97% percentage of identity with the reference sequence.

## Results

### Analysis of 18S rRNA

**Fig. 2:**
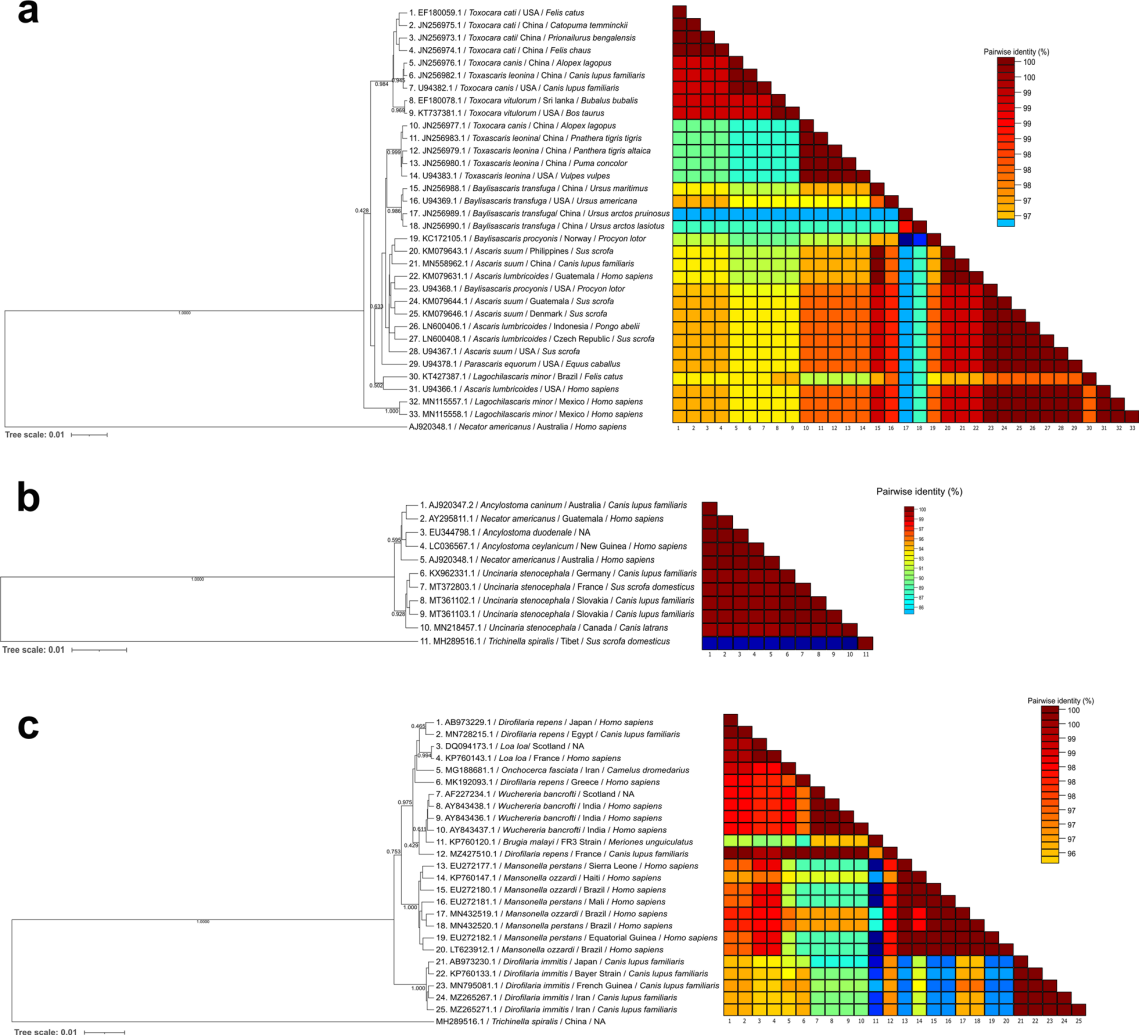
Phylogenetic Bayesian inference analysis of the nuclear 18S rRNA of selected species of veterinary and clinic importance of the families Ascarididae (**a**), Ancylostomatidae (**b**) and Onchocercidae (**c**). Nucleotide pairwise identity is denoted in the heatmap next to each taxa name

The analyses for the 18S rRNA region showed a minimum paired identity > 96.3% for the three studied families (Fig. 2). Accordingly, the lowest nucleotide similarity within Ascarididae species was 98.3%, in Ancylostomatidae was 99.60% and for Onchocercidae members was 96.3%.

The BI analysis revealed low posterior probabilities (PP) in the branches separating several species of the Ascarididae and Ancylostomatidae families, indicating sequences could not be resolved and separated with enough resolution. Therefore, the phylogenetic analysis with the 18S does not present a clear differentiation of the species of these families (Fig. 2a and b). When *Toxocara canis*, *T. cati* and *T. vitulorum* were compared, nucleotide similarities were > 99%, and all sequences retrieved from these species grouped in the same cluster with low PP in the inner ramifications. For the Ancylostomatidae family, only ten sequences could be obtained from GenBank, and these were divided in a tree branch containing the sequences of *Ancylostoma* spp. and *N. americanus*, while another tree branch contained *Uncinaria stenocephala* specimens (Fig. 2b), with low PP in inner ramifications. For the Onchocercidae, four main clusters were observed in the BI tree: one with *Dirofilaria repens*, *Loa loa* and *Onchocerca fasciata*; a second with *W. bancrofti*, *B. malayi* and a *D. repens* sequence from a dog from France; a third group with *Mansonella* spp.; a final cluster with *Dirofilaria immitis* sequences (Fig. 2c).

### Analysis of ITS-1 and ITS-2 ribosomal loci

**Fig. 3:**
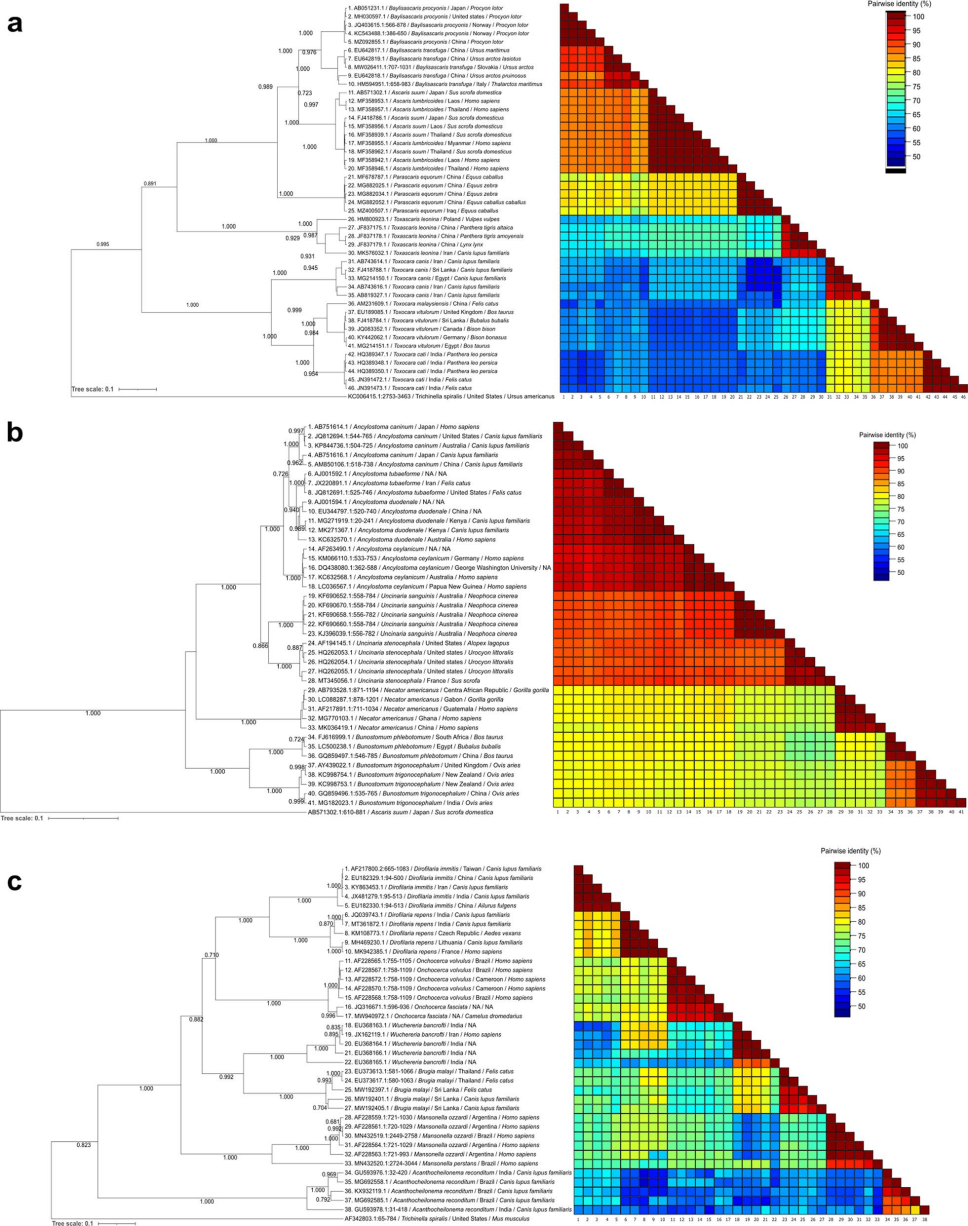
Phylogenetic Bayesian inference analysis of ITS2 loci of selected species of veterinary and clinic importance of the families Ascarididae (**a**), Ancylostomatidae (**b**) and Onchocercidae (**c**). Nucleotide pairwise identity is denoted in the heatmap next to each taxa name

The ITS regions showed high interspecies resolution with nucleotide pairwise identities > 51.2%. The ITS-1 region had an average minimum pairwise identity of 51.2%, 57.9% and 58.3% for the Ascarididae, Ancylostomatidae and Onchocercidae families, respectively, with complete separation of species in the BI trees (Additional file 4). Moreover, the ITS-2 showed 52.5% mean nucleotide pairwise similarity in the Ascarididae family, 72.8% for Ancylostomatidae and 51.5% in the Onchocercidae family (Fig. 3).

A total separation of species of the Ascarididae was observed in the ITS-1 and ITS-2 BI tree families, supported by PP values of 1.000 (Additional file 4 and Fig. 3). The ITS-1 and ITS-2 BI trees showed two clear clusters: one containing *Toxascaris leonina*, *Ascaris lumbricoides*, *A. suum*, *P. equorum*, *Baylisascaris procyonis* and *B. transfuga* sequences and a second containing *T. canis*, *T. cati* and *T. vitulorum* sequences. Remarkably, *A. lumbricoides* sequences from humans and *A. suum*, obtained from pigs and a human, clustered together with a minimum identity percentage of 98.4% for the ITS-1 and 100.0% for ITS-2. In addition, sequences of these two ascarids were intermixed in the cluster in the ITS-2 BI tree (Fig. 3a).

Four main groups could be identified in the ITS-1 and ITS-2 phylogenetic trees of the Ancylostomatidae family: one represented by *Ancylostoma* spp., a second represented by *Uncinaria sanguinis*, one represented by *Bunostonum* spp. and a group represented by *Necator americanus* sequences, all with PP = 1.000. Moreover, each species within each cluster was separated between them, showing a clear interspecies resolution.

For the Onchocercidae family, four sequence groups were observed in the BI tree of the ITS-1 region. Accordingly, one group contained *Mansonella* spp. sequences, the second contained *Onchocerca* spp., the third contained *W. bancrofti* and *B. malayi* and, lastly, a cluster contained *Dirofilaria* spp. and *L. loa* sequences (Additional file 4). Each species was separated with high nucleotide difference, except for *W. bancrofti* and *B. malayi*, whose differences were approximately 10%. ITS-2 phylogenetic tree built with sequences of this family exhibited the same clustering as ITS-1, with the addition of *Acanthocheilonema reconditum* sequences, which were located next to the *Mansonella* spp. group. Although four ITS-1 sequences of *A. reconditum* were available in GenBank, these did not meet the criteria to be included in the BI analysis.

### *cox*1 mitochondrial gene

**Fig. 4:**
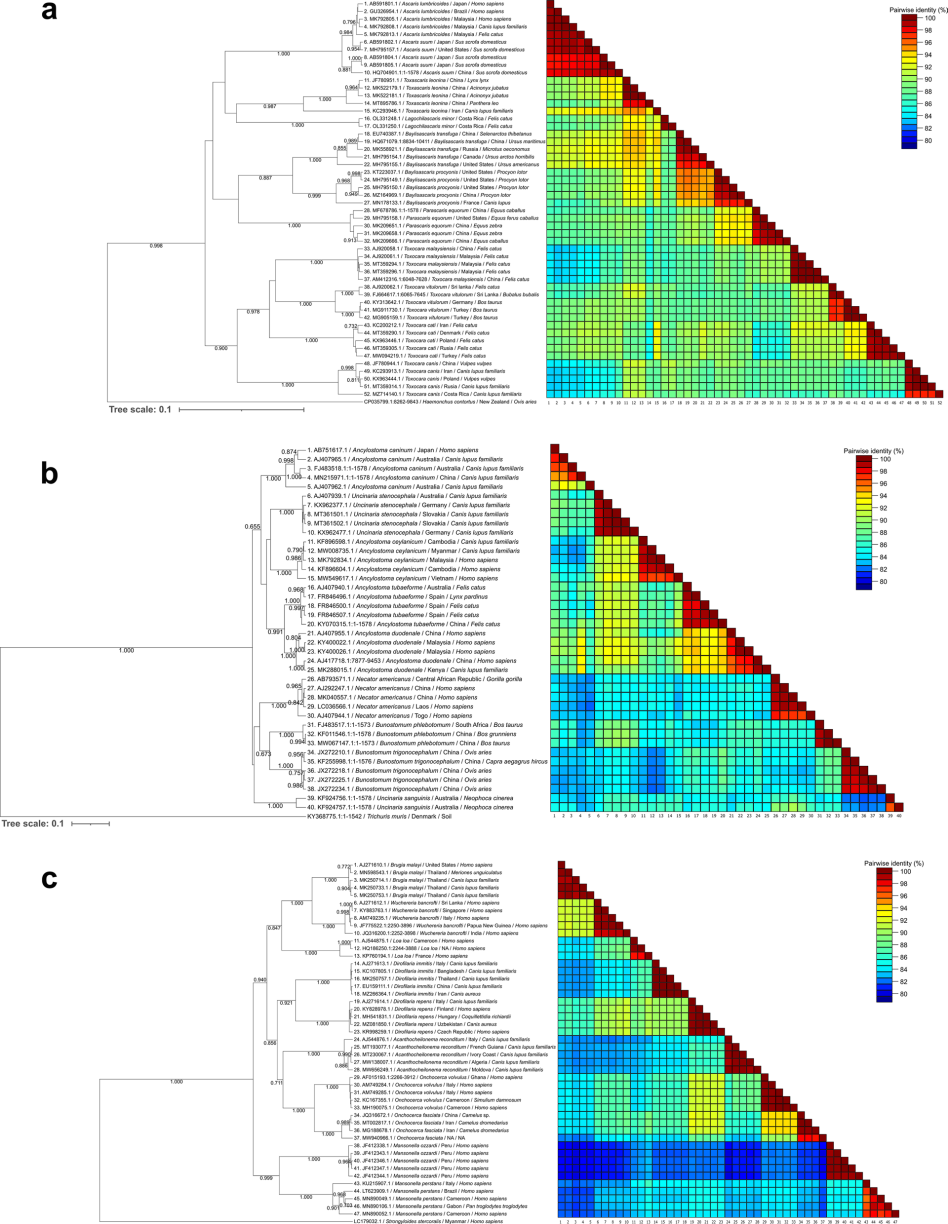
Phylogenetic Bayesian inference analysis of *cox*1 markers of selected species of veterinary and clinic importance of the families Ascarididae (**a**), Ancylostomatidae (**b**) and Onchocercidae (**c**). Nucleotide pairwise identity is denoted in the heatmap next to each taxa name

Average pairwise nucleotide similarities in the *cox*1 gene fragment within the Ascarididae, Ancylostomatidae and Onchocercidae families were 85.4%, 83.0% and 80.5%, respectively (Fig. 4). All species used in the analysis were successfully resolved in the phylogenetic tree for ascarids and showed a similar topology with the BI phylogenetic tree using the ITS-1 and ITS-2. Accordingly, three main clusters were observed: one with *A. lumbricoides*, *A. suum*, *T. leonina* and *Lagochilascaris minor*, a second group with *B. transfuga*, *B. procyonis* and *P. equorum* and a final one with *Toxocara* spp. Finally, as previously observed in the ITS-1 and ITS-2 trees, *A. lumbricoides* and *A. suum* clustered together, resulting in very high paired identity values, with the lowest value being 96.9% and the highest 100.0% (Fig. 4a). Moreover, two groups were observed in the Ancylostomatidae family tree, namely one with *Ancylostoma* spp. and *U. stenocephala* and another cluster with *N. americanus* and *Bunostomum* spp. (Fig. 4b). Phylogenetic analysis of the Onchocercidae sequences showed a marked divergence from *Mansonella* spp. regarding the other species of the family with high PP. In addition, *Acanthocheilonema reconditum* sequences were grouped with those of *Onchocerca* spp., in contrast to the topologies for the ITS-1 and ITS-2 trees, which were separated into different groups (Fig. 4c).

### 12S and 16S mitochondrial genes

Phylogenetic interspecies resolution was obtained in the analyzed 12S and 16S fragments (Additional files 5 and 6, respectively). The average pairwise nucleotide similarity in the 12S was 75.9% within the Ascarididae family, 84.7% for Ancylostomatidae and 81.5% within the Onchocercidae. On the other hand, the average pairwise nucleotide similarity for the 16S gene was 79.0%, 83.1% and 83.5% within the analyzed ascarid, ancylostomatid and onchocercid sequences. In addition, the number of sequences available for phylogenetic analysis for the 16S gene was lower than in the other loci.

Phylogenetic trees for 12S and 16S genes support the results previously obtained for *A. lumbricoides* and *A. suum*, which grouped together with a percentage of identity for the 16S sequences ≥ 97.0%. Furthermore, *A. lumbricoides*, *A. suum*, *P. equorum*, *B. procyonis*, *B. transfuga* and *T. leonina* were grouped in one of the branches of the tree, while *Toxocara* spp. sequences were clustered in a separate cluster with high PP and pairwise identities ranging from 77.0 to 79.0% compared to sequences of other ascarids.

In the 12S phylogenetic tree, two main branches could be observed, one with *N. americanus* sequences and the second with *Bunostonum* spp., *Ancylostoma*. spp. and *Uncinaria* spp. The tree topology for 16S sequences varied in that *Bunostomum* spp. sequences were separated from the other analyzed species, whereas *N. americanus* sequences were closer to *Ancylostoma* spp.

The phylogenetic trees of the 12S and 16S of the Onchocercidae sequences revealed a different topology to the one observed in the *cox*1 gene fragment. For the 12S tree, three groups were observed: a branch with *Onchocerca* spp. and *Dirofilaria* spp., a second group with *B. malayi*, *W. bancrofti*, *L. loa* and *Mansonella* spp., and a third cluster with *A. reconditum*. In the 16S phylogenetic tree, fewer sequences could be analyzed according to the inclusion criteria. Nevertheless, a group with *B. malayi*, *W. bancrofti*, *L. loa* and *O. volvulus* was obtained; a separate cluster was observed with *Dirofilaria* spp. and a single *M. perstans* sequence was derived from the tree. Therefore, *D. immitis* and *D. repens* were grouped together in both 12S and 16S genes, and the same was obtained with *B. malayi* and *W. bancrofti*.

### Nucleotide differences between genes of each family

**Fig. 5:**
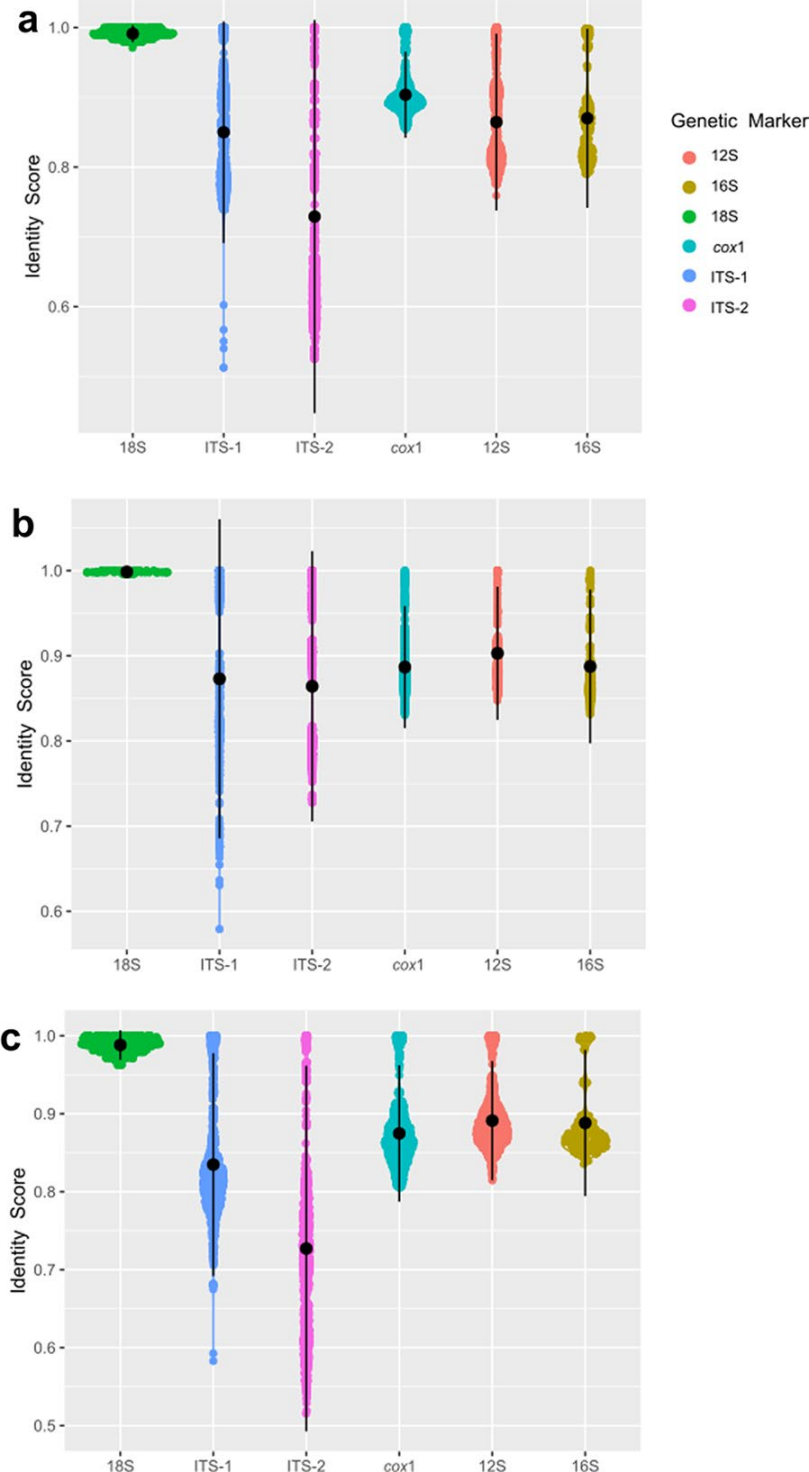
Nucleotide similarities between selected species of veterinary and clinic importance of the families Ascarididae (**a**), Ancylostomatidae (**b**) and Onchocercidae (**c**) in each marker analyzed in this study

Significant pairwise nucleotide p-distances in all markers were obtained for each family (all *P* < 0.0001, Kruskal-Wallis statistic = 2023, 208 and 1900 for Ascarididae, Ancylostomatidae and Onchocercidae families, respectively) (Fig. 5). A total of 4998 pairwise comparisons were calculated for the Ascarididae family, 2828 for the Ancylostomatidae family and 4038 for the Onchocercidae family.

The highest pairwise nucleotide similarity in the Ascarididae family was shown in the nuclear rRNA 18S fragment with an average of 99.1 ± 0.6%, which was significantly different from the other five markers (all *P* < 0.01) (Fig. 5a). Average pairwise nucleotide p-distances of the analyzed *cox*1 fragments were 90.4 ± 3.1%, whereas the pairwise nucleotide similarities in the 16S, 12S, ITS-1 and ITS-2 markers were 87.0 ± 6.4%, 86.4 ± 6.3%, 85.0 ± 7.9% and 72.9 ± 14.1%, respectively. Significant differences were detected when comparing the values of each marker between them, except between average pairwise p-distances of 12S and 16S, 16S and ITS-1, and 12S and ITS-1.

For the Ancylostomatidae family, the highest pairwise similarity was obtained in the 18S fragment, with an average of 99.8 ± 0.1% similarity between sequences, followed by the 12S with 90.3 ± 3.9%, the 16S with 88.7 ± 4.5%, *cox*1 with 88.7 ± 3.6%, ITS-1 with 87.3 ± 9.4% and ITS-2 with 86.4 ± 7.9% average pairwise similarities (Fig. 5b). The average pairwise p-distance was significantly different when comparing the data of the 18S to the other five markers with all *P* < 0.01. Other pairwise statistical comparisons were significant except between data of 16S and *cox*1, *cox*1 and ITS-1, and ITS-1 and ITS-2, whose average pairwise distance where similar between them.

Similar results to those obtained for the Ascarididae and Ancylostomatidae families were evident for the Onchocercidae family (Fig. 5c), with the nuclear rRNA 18S showing the highest pairwise nucleotide similarities (98.8 ± 0.9%) with significant difference (all *P* < 0.01) compared to the 12S (89.1 ± 3.8%), 16S (88.8 ± 4.7%), *cox*1 (87.5 ± 4.4%), ITS-1 (83.5 ± 7.1%) and ITS-2 (72.7 ± 11.7%). Significant differences were obtained in all pairwise comparisons of loci except between the 16S and 12S and the 16S and *cox*1.

### Availability of sequences in the GenBank database

**Fig. 6:**
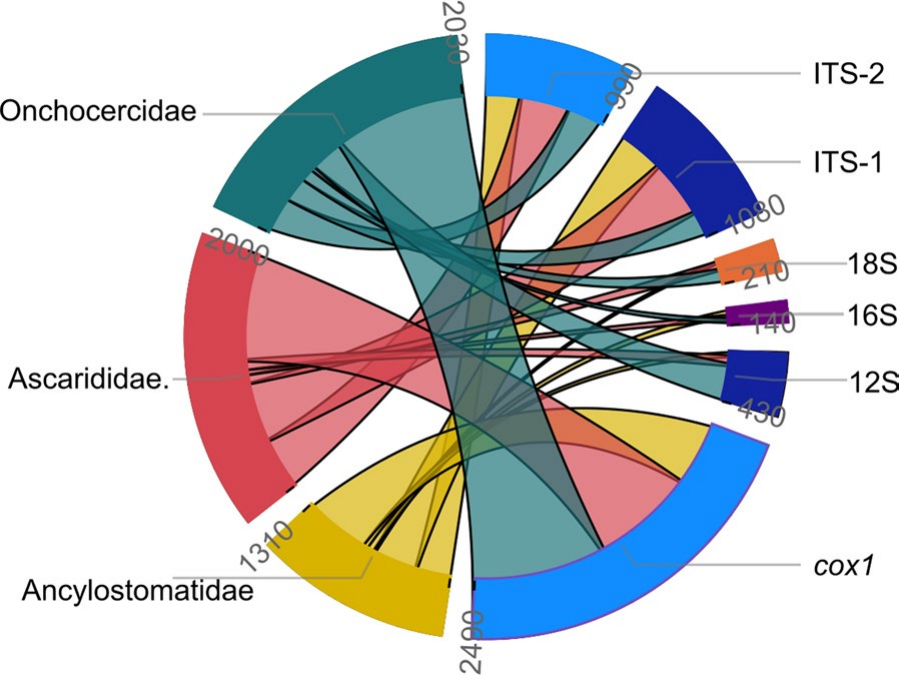
Availability of *cox*1, 12S, 16S, ITS-1, ITS-2 and 18S sequences of selected species of veterinary and clinical importance of the families Ascarididae, Ancylostomatidae and Onchocercidae in NCBI databases. Circular Sankey chart of the number of sequences available for each marker depending on the family. Sum of sequences available for each marker by family

The marker with the highest representation in GenBank was the *cox*1 with a total of 2491 sequences for the evaluated species, followed by the ITS-1 (*n* = 1082), ITS-2 (*n* = 994), 12S (*n* = 428), 18S (*n* = 212) and 16S (*n* = 143) (Fig. 6). Complete results with the number of sequences per analyzed species is available in Additional file 7. Accordingly, the analyzed species of the Ascarididae family had 870 *cox*1 sequences deposited in GenBank, whereas 525 sequences were available for the species of the Ancylostomatidae family and 1096 for the Onchocercidae family. For the ITS-1, 498, 375 and 209 sequences were retrieved from GenBank in the studied species of the Ascarididae, Ancylostomatidae and Onchocercidae families, respectively. The marker with the least sequence availability was the 16S mitochondrial rRNA gene with 46 sequences in the analyzed species of the Ascarididae family, 56 for the Ancylostomatidae and 41 for the Onchocercidae. The numbers of sequences per species are presented in Table 1.

**Table 1:** Number of sequences retrieved from GenBank of nematodes of veterinary and clinical importance of the Ascarididae, Ancylostomatidae and Onchocercidae families

Family	Species	18S	ITS1	ITS2	*cox*1	12S	16S
Ascarididae	*Ascaris lumbricoides*	7	129	27	238	18	17
	*Ascaris suum*	17	44	19	114	12	7
	*Toxascaris leonina*	9	66	62	76	13	5
	*Baylisascaris transfuga*	4	15	19	25	10	2
	*Baylisascaris procyonis*	4	47	13	165	4	2
	*Parascaris equorum*	1	49	53	29	4	3
	*Toxocara vitulorum*	4	17	15	7	6	1
	*Toxocara malaysiensis*	0	0	1	9	2	2
	*Toxocara cati*	17	82	135	93	6	2
	*Toxocara canis*	12	49	75	103	11	5
	*Lagochilascaris minor*	5	0	0	11	2	0
Ancylostomatidae	*Ancylostoma caninum*	2	181	96	52	4	4
	*Ancylostoma tubaeforme*	0	22	3	20	2	2
	*Ancylostoma duodenale*	1	15	5	23	3	6
	*Ancylostoma ceylanicum*	1	67	58	128	4	3
	*Uncinaria sanguinis*	0	22	22	59	3	3
	*Uncinaria stenocephala*	16	0	24	21	15	5
	*Bunostomum phlebotomum*	0	1	3	4	4	4
	*Bunostomum trigonocephalum*	0	5	8	41	3	3
	*Necator americanus*	4	62	72	177	5	26
Onchocercidae	*Brugia malayi*	3	62	36	17	7	3
	*Wuchereria bancrofti*	5	7	5	499	7	5
	*Loa loa*	2	19	0	3	5	1
	*Dirofilaria immitis*	61	34	118	194	124	9
	*Dirofilaria repens*	25	4	31	319	114	7
	*Acanthocheilonema reconditum*	0	1	45	25	15	0
	*Onchocerca volvulus*	0	13	18	8	14	13
	*Onchocerca fasciata*	1	2	2	4	5	2
	*Mansonella ozzardi*	4	13	28	10	4	0
	*Mansonella perstans*	7	54	1	17	2	1

## Discussion

We analyzed the interspecies resolution and sequence availability of different mitochondrial and ribosomal markers commonly used for diagnosing helminthiases of medico-veterinary significance. The present work determined that the mitochondrial gene *cox*1 had the highest number of available sequences in GenBank, indicating this is a suitable marker to use when confirming the identity of an unknown specimen since many sequences are available for comparison. Moreover, this analysis included sequences from different hosts and geographical regions, thus giving a wider perspective of the genetic variability of each species. The nuclear rRNA 18S gene showed the lowest species resolution, whereas nuclear ITS-1 and ITS-2 regions showed the highest nucleotide differences between species, followed by mitochondrial gene *cox*1 and mitochondrial rRNA genes 12S and 16S. Furthermore, the *cox*1 gene was the most represented marker in GenBank, with almost the same number of sequences of 18S, ITS-1, ITS-2, 12S and 16S summed together for the analyzed species.

The results obtained for the 18S gene of the Ascarididae, Ancylostomatidae and Onchocercidae families indicate that this marker does not separate between close species, since nucleotide similarity values between 98.8 and 99.9% were obtained. The analyzed species were intermixed in the phylogenetic trees of the three families, especially between *A. lumbricoides* and *A. suum*, *Toxocara* spp. with *T. leonina*, *W. bancrofti* and *B. malayi*, and among *Mansonella* spp. Therefore, this marker may resolve species at higher taxonomic levels [15, 22, 23], which may be useful for describing nematode communities in soil [24, 25] or biological animal samples [26] using 18S-based metagenomics. However, it does not provide the necessary resolution for identifying unknown specimens in a clinical setting [27]. For instance, an unknown specimen may be erroneously classified if only an 18S fragment is analyzed, such as the case of *L. minor* from cats, which showed 99% homology to *A. suum* [27]. For these cases, the analysis of several and longer DNA fragments is recommended, together with the close morphological examination of specimens. The low interspecies difference in the 18S may be explained by the concerted evolution of this coding region, which homogenizes all copies in a specimen’s genome [28] and secures the structure of rRNA functional domains, as opposed to the non-coding ITS regions [29].

The ITS-1 and ITS-2 regions yielded the highest percentage of sequence variability and separated all species in phylogenetic analyses except *A. suum* and *A. lumbricoides*, which were intermixed and *Ancylostoma* spp. that showed lower nucleotide differences between them. Although the ITS regions are part of the nuclear rRNA operon, these loci have a higher nucleotide substitution rate than the 18S [29], which eases differentiation at the species level [15]. This characteristic was confirmed in the present study and has been observed for nematodes of other families like Spirocercidae [30], Strongylidae [31] and Trichuridae [32], to name some examples. Nucleotide differences in these loci are so high that they may even exhibit a high genetic intra-individual variation, i.e. a single specimen with different copies of ITS, such as the case of the dog carcinogenic nematode *Spirocerca lupi* [30], the whipworms *Trichuris* spp. [33] or the causing agent of cystic echinococcosis, *Echinococcus granulosus* [34]. In this study, ITS-1 and ITS-2 proved to be useful markers in helminth identification applications. Nevertheless, the high nucleotide difference between species difficulted subsequent analyses, such as sequence alignment and phylogenetic reconstructions when using large datasets [15]. For this reason, ideal diagnostic markers would be those showing enough interspecies nucleotide differences but easy to handle for beginners.

The three markers of mitochondrial origin analyzed in this work had a similar performance in terms of their sequence resolution in phylogenetic analyses and the pairwise nucleotide differences. It has been argued that mitochondrial rRNA 12S and 16S are less variable than *cox*1. However, we did not observe significant statistical differences in the percentage of nucleotide variation in the three markers [15]. The *cox*1 gene has been used in DNA barcoding and is currently considered the universal genetic marker for this application [35]. DNA barcoding uses a small consensus DNA fragment of the *cox*1 to globally identify a large number of organisms from different geographical locations, hosts or genetic subclassifications [35–37]. Mitochondrial markers have several advantages compared to those of nuclear origin. For example, mitochondrial markers do not undergo recombination and have a higher mutation rate than nuclear rRNA 18S, 5.8S or 28S [38]. Therefore, mitochondrial genes are useful in resolving organisms at lower taxonomic levels to the species level and thus may be useful for metabarcoding purposes.

Although mitochondrial markers have great variability, it has been argued that the design of universal primers targeting mitochondrial regions may be challenging [39]. However, previous studies have successfully amplified consensus *cox*1 sequences from different nematode families [40] and have described free-living marine nematode communities [41]. Moreover, a study of seven nematode genera across different families showed an interspecies mean p-distance or nucleotide difference in the *cox*1 between 10 and 25%, whereas intraspecific pairwise nucleotide differences ranged from 0.5 to 3.5% [39]. Those results resemble the ones obtained in the present study. Altoghether, our results support the applicability of *cox*1 for metabarcoding studies.

The number of *cox*1 sequences available in GenBank exceeded those of the other five markers according to the employed mining pipeline. The present study analyzed this aspect for the first time, thus giving a different perspective regarding the usefulness of each locus in a diagnostic context. The larger number of *cox*1 sequences may be explained by the high sensitivity of the primers used for amplifying this gene, the widespread use of certain primers targeting the *cox*1 and the efforts of research groups worldwide in using and depositing barcodes of their specimens, thus including all possible biodiversity [39]. Interspecies resolution obtained in the 12S and 16S rRNA mitochondrial genes were comparable to those of the *cox*1 and therefore may have similar utility in metabarcoding analyses. However, the use of 12S and 16S and their applicability for phylogenetic, taxonomic and barcoding analysis would be limited by the low number of sequences in databases. A similar situation was observed with ITS regions, which provide larger nucleotide differences between species compared to nucleotide markers as obtained before [15], but their lower availability would limit their use for diagnostic or metabarcoding purposes. Therefore, sequencing and deposition of *cox*1, 12S, 16S, ITS-1 and ITS-2 sequences from nematode specimens are strongly encouraged to include the most comprehensive genetic information, which will be useful in future studies.

Interestingly, *A. lumbricoides* and *A. suum* sequences were intermixed in all analyzed markers, thus raising the long-standing question of whether these two taxonomic units actually represent the same species [42]. Cryptic diversity is defined as two morphologically indistinguishable but genetically different entities [43]. Additionally, other parameters have been used to infer a cryptic species including host preference, geographic distribution, resource use and reproductive behavior [18]. Accordingly, both ascarids have been proposed as cryptic species since *A. suum* was named as a pig roundworm by Goeze (1782), even though it did not present any morphological differences from *A. lumbricoides*. The present study showed that *A. suum* and *A. lumbricoides* clustered together in the phylogenetic trees of the different genes and had a pairwise nucleotide identity between 97 and 100%. In fact, genomic analyses of *A. lumbricoides* obtained from humans of a village without pig husbandry showed a high heterozygocity and hybridization of *A. lumbricoides* and *A. suum,* suggesting a potential interbred *Ascaris* complex infecting humans [44]. In any case, an allele-specific PCR using the ITS-1 of *A. suum* and *A. lumbricoides* showed good resolution for discriminating both ascarids, which may become useful for epidemiological and control purposes [45].

The selection of markers used in the present study was not exhaustive, and they were chosen according to their current use for diagnostic purposes. Other mitochondrial genes such as the *nad*1 and *cyt*b have been evaluated before [15], rendering similar interspecies variation as obtained herein for *cox*1, 12S and 16S analyses. Additionally, representative nuclear and mitochondrial sequences of nematodes, trematodes and cestodes of clinic and veterinary importance have been evaluated before. Nevertheless, we focused on 30 species of three nematode families of medico-veterinary importance, rendering up to five sequences from each species when available. In this way, genetic variation arising from geographic or host origin could be included herein, which has not been studied before to our knowledge. Importantly, these kind of studies are necessary to guide basic and medical researchers in their decision-making when unknown worms arrive at their laboratories or clinics.

Even though molecular diagnosis of nematodes may be objective and straightforward [46], a proper nematode identification should include morphological analysis of specimens [14]. Molecular data complement morphological observations of worms, which may lead to the identification of cryptic diversity sensu lato or sensu stricto, and the reclassification of certain taxa or the description of new species [18]. This integrative taxonomic approach has been suggested before for filarioids [14] and has proven essential for the description of the true biodiversity of a genus, family or phyla. Furthermore, incorrect deposition of genetic data has been demonstrated in other invertebrate orders [47] and for the dyphyllobothridean cestodes of the genus *Spirometra* [48]. This highlights the responsibility of researchers in the selection of methods and the interpretation of their results.

## Conclusions

Phylogenetic relationships and minimal pairwise nucleotide identities were analyzed in six different molecular markers from 30 species of the Ascarididae, Ancylostomatidae and Onchocercidae families. Importantly, we assessed, for the first time to our knowledge, the availability of those sequences in databases as a complement for choosing a molecular marker in a clinical or research context. Results showed that *cox*1 is a useful marker for diagnostic or metabarcoding purposes due to the high interspecies resolution demonstrated in phylogenetic analyses and the higher number of sequences available in databases. This will enable the accurate identification of an unknown specimen when comparing their associated sequences to those deposited in GenBank. Finally, we recommend the combined analysis of ITS regions, other mitochondrial genes and morphological analysis of specimens whenever possible.

### Supplementary Information

**Additional file 1: Table S1.** Accession numbers of sequences used in the study.

**Additional file 2: Table S2.** Best nucleotide substitution models for each marker and family used in the analysis.

**Additional file 3. **Script used for constructing violin plots with nucleotide p-distances.

**Additional file 4. **Phylogenetic Bayesian inference analysis of ITS1 loci of selected species of veterinary and clinic importance of the families Ascarididae (a), Ancylostomatidae (b) and Onchocercidae (c). Nucleotide pairwise identity is denoted in the heatmap next to each taxa name.

**Additional file 5. **Phylogenetic Bayesian inference analysis of 12S genes of selected species of veterinary and clinic importance of the families Ascarididae (a), Ancylostomatidae (b) and Onchocercidae (c). Nucleotide pairwise identity is denoted in the heatmap next to each taxa name.

**Additional file 6. **Phylogenetic Bayesian inference analysis of 16S loci of selected species of veterinary and clinic importance of the families Ascarididae (a), Ancylostomatidae (b) and Onchocercidae (c). Nucleotide pairwise identity is denoted in the heatmap next to each taxa name.

**Additional file 7. **PowerBI dashboard of 18S rRNA, ITS-1, ITS-2, *cox*1, 12S and 16S sequences available in GenBank for 30 species of the Ascarididae, Ancylostomatidae and Onchocercidae families. https://app.powerbi.com/view?r=eyJrIjoiZGNhN2MwY2EtYjI1OS00NjFlLWIzZWItMTk0NGIzMzA1NTM2IiwidCI6ImU3OTg0Y2FjLTI1NDMtNGY4OC04Zjk3LTk1MjQzMzVlNmJjNCIsImMiOjR9.

## Data Availability

Analysis of sequence availability is publicly available at https://app.powerbi.com/view?r=eyJrIjoiZGNhN2MwY2EtYjI1OS00NjFlLWIzZWItMTk0NGIzMzA1NTM2IiwidCI6ImU3OTg0Y2FjLTI1NDMtNGY4OC04Zjk3LTk1MjQzMzVlNmJjNCIsImMiOjR9.

## Abbreviations

*cox*1: Cytochrome oxidase subunit 1
ITS: Internal transcribed spacer
