# Supplementary figures and images for: Mitochondrial and ribosomal markers in the identification of nematodes of clinical and veterinary importance
Source: Parasit Vectors. 2024 Feb 20;17:77. doi: 10.1186/s13071-023-06113-4 (PMC10880205; doi:10.1186/s13071-023-06113-4)

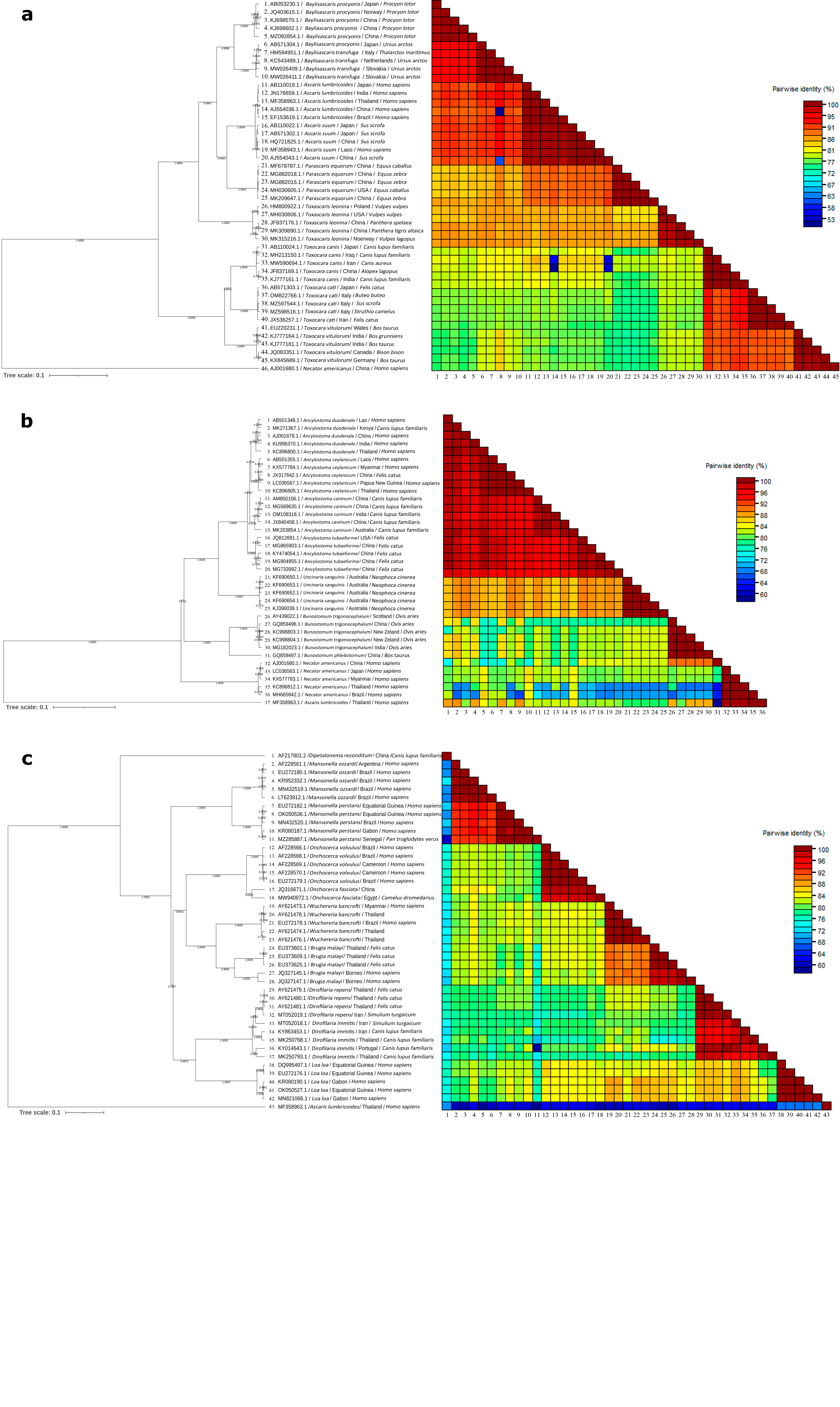

Supplement: Supplementary file 4 — Additional file 4. Phylogenetic Bayesian inference analysis of ITS1 loci of selected species of veterinary and clinic importance of the families Ascarididae (a), Ancylostomatidae (b) and Onchocercidae (c). Nucleotide pairwise identity is denoted in the heatmap next to each taxa name. [file 13071_2023_6113_MOESM4_ESM.png]

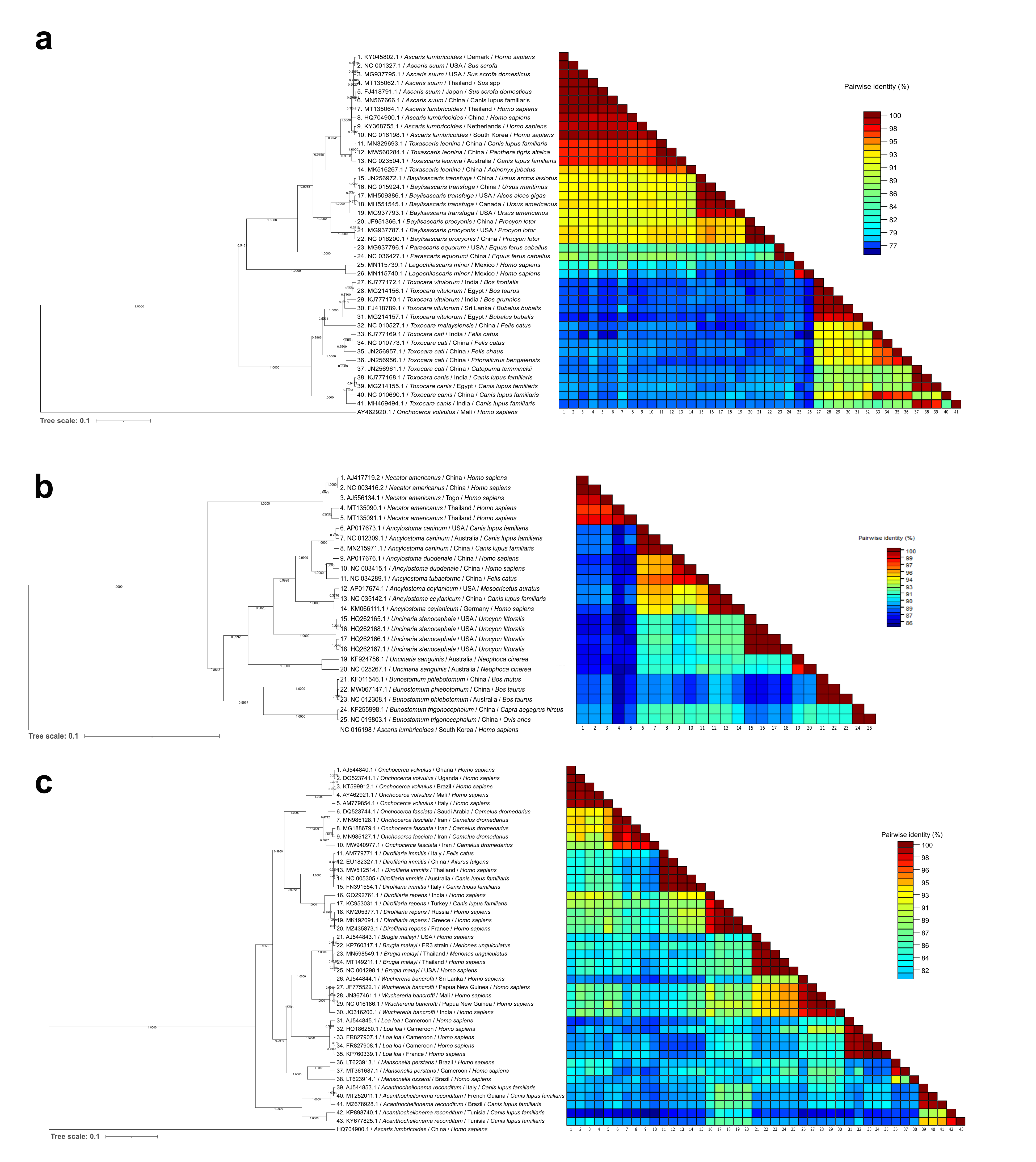

Supplement: Supplementary file 5 — Additional file 5. Phylogenetic Bayesian inference analysis of 12S genes of selected species of veterinary and clinic importance of the families Ascarididae (a), Ancylostomatidae (b) and Onchocercidae (c). Nucleotide pairwise identity is denoted in the heatmap next to each taxa name. [file 13071_2023_6113_MOESM5_ESM.png]

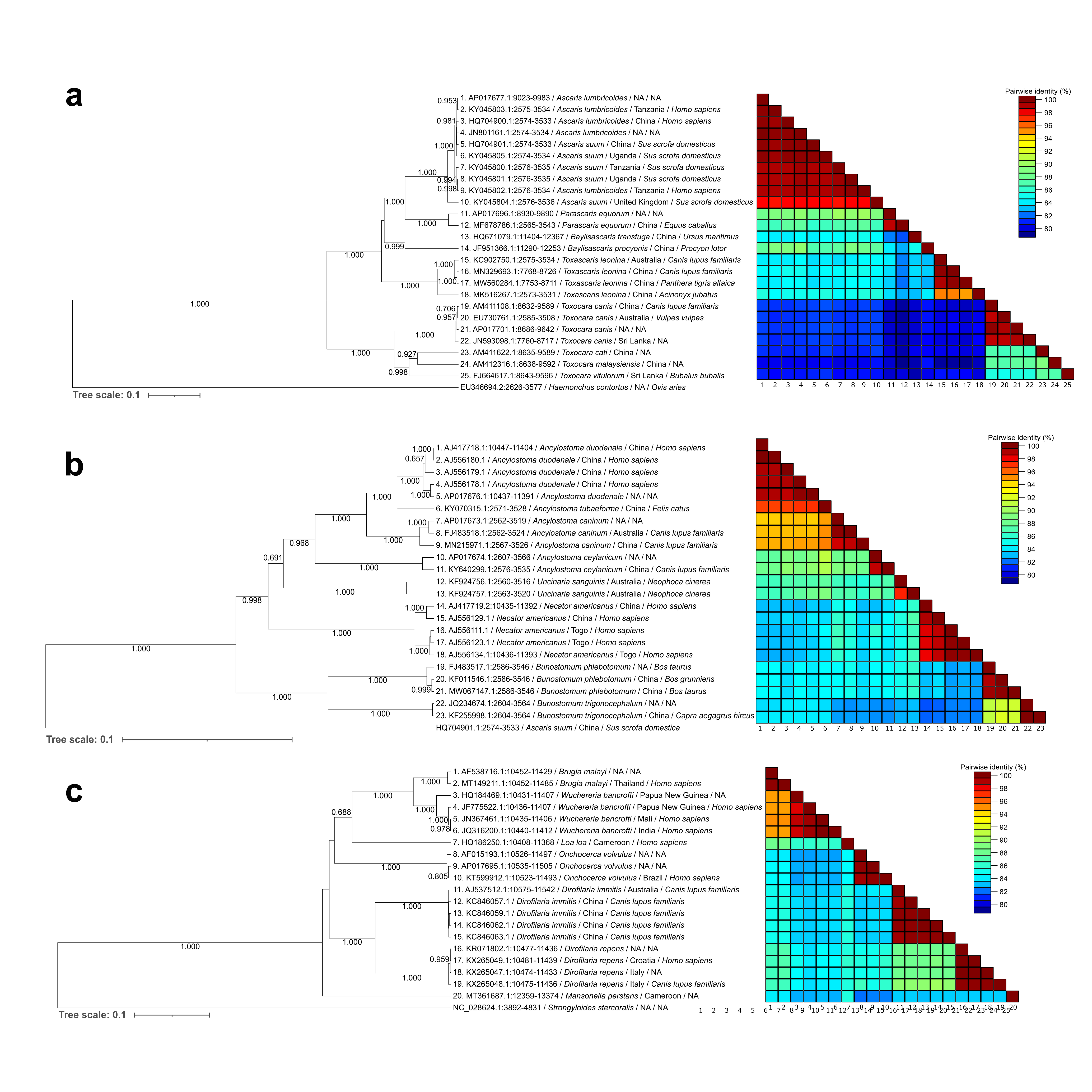

Supplement: Supplementary file 6 — Additional file 6. Phylogenetic Bayesian inference analysis of 16S loci of selected species of veterinary and clinic importance of the families Ascarididae (a), Ancylostomatidae (b) and Onchocercidae (c). Nucleotide pairwise identity is denoted in the heatmap next to each taxa name. [file 13071_2023_6113_MOESM6_ESM.png]
