# Supplementary material for: Mitochondrial and ribosomal markers in the identification of nematodes of clinical and veterinary importance
Source: Parasit Vectors. 2024 Feb 20;17:77. doi: 10.1186/s13071-023-06113-4 (PMC10880205; doi:10.1186/s13071-023-06113-4)
Supplement: Supplementary file 7 — Additional file 7. PowerBI dashboard of 18S rRNA, ITS-1, ITS-2, cox1, 12S and 16S sequences available in GenBank for 30 species of the Ascarididae, Ancylostomatidae and Onchocercidae families. https://app.powerbi.com/view?r=eyJrIjoiZGNhN2MwY2EtYjI1OS00NjFlLWIzZWItMTk0NGIzMzA1NTM2IiwidCI6ImU3OTg0Y2FjLTI1NDMtNGY4OC04Zjk3LTk1MjQzMzVlNmJjNCIsImMiOjR9. [file 13071_2023_6113_MOESM7_ESM.docx]

**Additional file 7.** PowerBI dashboard of 18S rRNA, ITS-1, ITS-2, *cox*1, 12S and 16S sequences available in GenBank for 30 species of the Ascarididae, Ancylostomatidae and Onchocercidae families. [https://app.powerbi.com/view?r=eyJrIjoiZGNhN2MwY2EtYjI1OS00NjFlLWIzZWItMTk0NGIzMzA1NTM2IiwidCI6ImU3OTg0Y2FjLTI1NDMtNGY4OC04Zjk3LTk1MjQzMzVlNmJjNCIsImMiOjR9](sps:urlprefix::https)
